# Cancer care in Lebanon and the climate-proofing of a burning phoenix

**DOI:** 10.3332/ecancer.2023.ed130

**Published:** 2023-11-09

**Authors:** Mohamad Ibrahim

**Affiliations:** The Royal Marsden, Fulham Road, London SW3 6JJ, UK

**Keywords:** Lebanon, healthcare system, cancer care, climate proofing, climate change, economic crisis

## Abstract

Beirut, the capital of Lebanon, has been burnt and rebuilt seven times, earning the title of an urban phoenix. Its strategic geolocation in the heart of the Mediterranean gave the country leverage for the economy, by becoming a hotspot for trade through its different ports. Its cultural and religious diversity provided a fertile ground for businesses to grow, most importantly the banking and healthcare sector. The strategic location also put the country in the eye of the storm of different major crises, leaving the small country a crippling state, swamped with political corruption, nepotism, fragile infrastructure, and an overall dysfunctional system. In October 2019, the collapse of the banking system led to a state of hyperinflation in the country, with the financial and economic crisis in Lebanon now ranked in the top 10 most severe crises episodes globally since the mid-nineteenth century. This crisis exposed the extent of corruption and dysfunctionality: a fragmented healthcare sector struggling to protect the vulnerable populations especially cancer patients, a workforce seeking exodus due to challenging work conditions amid a state of hyperinflation, and a fragile infrastructure unable to withstand climate changes in one of the hottest and driest areas on the planet, the Middle East. Despite being caught in this turmoil, Lebanon still managed to update its national climate pledge under the Paris Agreement. However, the financial recovery plan that is aiming to revive the economy and overcome short-term financial challenges, has a risk of affecting long-term climate-proofing efforts. In a multilevel crisis-torn country, it might be difficult to look on the impact of climate change in particular on cancer care, without accounting for the other ongoing troubles. In such circumstances, protecting public health becomes a major challenge yet a priority. The resilience of the system is currently being tested as the crisis evolves, and should be continuously monitored.

## Lebanon: a country amid storms

Tales have it that Beirut, the capital of Lebanon, has been burnt and rebuilt seven times, earning the title of an urban phoenix [1]. Lebanon is one of the smallest middle eastern countries, with a size of 10452 sq. Km, roughly around 4.3% of the size of the United Kingdom [2]. Its strategic geolocation in the heart of the Mediterranean, gave the country leverage for the economy, by becoming a hotspot for trade through its different ports via land, sea, and air. Its cultural and religious diversity provided a fertile ground for businesses to grow, most importantly the banking and healthcare sector. In its golden years, the country became known as the ‘Switzerland of the Middle East’, a description referring to both countries’ banking secrecy laws and snow-covered mountains.

However, the strategic location also put the country in the eye of the storm of different major crises, most notably the ongoing Arab-Israeli conflict, leading eventually to the Lebanese-Palestinian conflict, and triggering the Lebanese civil war that ran from 1975 to 1990. Most recently, the Syrian civil war (2011-ongoing) pushed an influx of over a million refugees towards Lebanon, becoming the country with the highest number of refugees per capita worldwide [3]. These events left the small country in a crippled state, swamped with political corruption, nepotism, fragile infrastructure, and an overall dysfunctional system, which failed to preserve the country’s assets, the public, and the different sectors from being affected by the surrounding.

## The healthcare system in Lebanon

The healthcare system in Lebanon managed to hold its ground despite the ongoing turmoil, with better services being provided through partnerships of the public and private sector. Health coverage in Lebanon relies mostly on the National Social Security Fund (NSSF), other governmental bodies such as the civil servants cooperative or the military schemes, or by private insurance, with almost one half of the population covered by either [4]. This approach led to a major reliance on private hospitals and insurers, with 80% of hospitals in the country being privately owned [4]. the private sector was booming, stimulating medical tourism from neighbouring countries.

Several governmental efforts were put through the Lebanese Ministry of Public Health (MoPH), in collaboration with non-governmental organisations (NGOs), to protect the public amid the ongoing varied struggles; Primary healthcare centres are operated mainly by NGOs, with almost 68% of centers being owned by NGOs through agreements with MoPH within the national network [4]. Syrian refugees are able to access subsidised services through coverage from United Nations High Commissioner for Refugees (UNHCR). Palestinian refugees can access similar services through the coverage the United Nations Relief and Work Agency for Palestine Refugees in the Near East (UNRWA) [4]. Vulnerable Lebanese populations are also capable of accessing essential primary care services and non-communicable diseases through the subsidisation provided by different NGOs. However, this contributed to the further fragmentation of a majorly privatised sector.

## Cancer care

For cancer care, the government established the ‘National Cancer Register’ in 2002 [5], which aimed to measure incidence and classification by type, age and sex, for example [6]. The ministry was able to provide oncology drugs free of charge since 1999 for all cancer patients in the country with no other formal health coverage [7], and ran some successful campaigns such as the yearly breast cancer screening campaigns. However, the focus remained on tertiary care and minimal efforts were put on early detection and prevention. Today, Lebanon has the highest incidence of cancer among the Arab countries [6].

## The economic crisis of 2019

In October 2019, protests around the country were triggered by a governmental proposal to increase taxes, amid increasing national financial struggles. For the first time in decades, protests took place on a national level, swamping cities from South to North. A country heavily divided by sectarianism was for a moment unified again through common struggles. Shortly after, the banking system started to crumble: the country was drained of its reserves of US dollars, a crucial asset for the economy that was built around pegging the Lebanese pound against the US dollars since 1997 [8], a financial model praised during its brief period of success, yet now described as a catastrophic nationally regulated Ponzi scheme. The political instability driving tourists away, along inefficient governmental spending and ongoing corruption, led to the fleeing of the capital from the country, and drained its existing dollars. Banks failed to give people back their deposits, limitations were put on amounts of money that can be accessed, currency started devaluating in the black market (lost 90% of its value to date), and inflation engulfed the country. In spring of 2021, the World Bank declared that Lebanon is in a state of hyperinflation, stating that the financial and economic crisis in Lebanon is likely to rank in the top 10, possibly top three, most severe crises episodes globally since the mid-nineteenth century [9]. The collapse of October 2019 was aggravated by the events that followed shortly after: the COVID-19 pandemic hitting on March 2020, and the explosion of the port of Beirut in August of the same year, one of the biggest non-nuclear explosions in history. The country was in a perfect storm. The phoenix was burning once again.

## Post-crisis struggles

### Healthcare system

Within said circumstances, it was only a matter of time before all sectors in the country started to crumble. The healthcare sector, already fragmented, mostly privatised, and overwhelmed by the influx of refugees, found itself facing a global pandemic, amid a state of bankruptcy, and an explosion that left behind over 6000 injured and several hundred permanent disabilities. Vulnerable populations became even more vulnerable, and new groups became vulnerable as the poverty line increased. Accessing healthcare services grew to become a luxury that only those with remaining US dollars, *fresh dollars^1^,* could afford. Cancer patients were about to be hit by the storm.

By 2021, MoPH had to reduce its subsidizations of most essential services and medications, including cancer treatments. Since the country relies mostly on imported medications, severe medicine shortages were unavoidable, and trading of medications on the black market followed. With citizens unable to access their own money in banks due to withdrawal limits, the status-quo made the unaffordable private care even further unreachable. The wealthiest in the country are still able to get access to medications from abroad or over the black market. However, some people are finding themselves having to go to extreme measures sometimes, taking matters into their own hands. Last September 2022, a Lebanese woman hit the headlines for holding a bank branch at gunpoint, forcing its employees to withdraw her own deposits which were unreachable at this point, given the existing limitations. She declared that the move was to pay for her sister’s cancer treatment [10]. Bank employees acquiesced and the woman left with a bag of cash. Public opinion was divided on the matter, as ethical as it might sound, it was also normalising chaos. Unsurprisingly, the country woke up the next day to at least eight banks held up by frustrated depositors, forcing the banks to go into a national strike [11].

### Healthcare workers exodus

With a collapsed economy, and crumbling sectors, the workforce was the next piece to catch the flame. The salary of a nurse dropped from an average of 700 USD/month in 2019, to almost 50 USD in 2020, due to the hyperinflation and currency devaluation. Working with a shortage of supplies, medications, power-cuts, within a global pandemic, rising cancer rates, while being under-paid, in a profession that is already challenging, the migration of skilled nurses, and skilled workers overall became an inevitable doom. In September 2021, WHO estimated that around 30% of nurses and 40% of doctors have left the country since October 2019, seeking better opportunities abroad [12]. Today, these numbers are expected to be even higher. Devastating statistics for professions that already struggle with recruitment and retention. Departing staff mean even further less available services.

### Climate change effects

The last piece in this dystopian burning phoenix scenario, is an actual fiery scene. The country is in the Middle East, one of the hottest and driest areas on the planet, where climate change is already doing grave damage to its inhabitants. Wildfires spread significantly across the country and the region in 2019 and 2020. The world witnessed a record of the warmest year in 2016, with the second warmest occurring shortly after in 2020 [13].

Global warming is having a detrimental impact on the planet, where sea levels are rising, the polar ice sheets and glaciers are melting, and extreme weathers are leading to severe droughts, floodings, and wildfires. Lebanon is at a particularly high risk for coastal zones floodings and desertification. Years of corruption and a major ongoing energy crisis has meant that no significant effort has been made in building any climate resilience or work towards green energy. The main water sources in the country are heavily polluted by untreated sewage, use of fertilisers and pesticides, landfilling, and improper disposal of solid waste.

Globally, climate change events are having a major impact on health, with predictions of over half a million deaths worldwide by 2050, including deaths from cancer [14]. Burning of fossil fuels increases level of carbon monoxide, leading to an increase in lung cancer and premature mortality [15]. Extreme weathers are affecting food productions and pushing intensive farming using carcinogenic pesticides [14]. Healthy diets are becoming more and more difficult to achieve, increasing the risks of cancers. Increased exposure to ultraviolet radiation is linked to a rise in all skin cancers [13]. For Lebanon, these projections could be even worse.

## Recovery plans and climate-proofing

A collapsed economy, devaluating currency, skilled workers exodus, climate change, rising pollution, increasing cancer rates: the situation in Lebanon seems bleak. Yet, if there is one thing that Lebanese people have learnt throughout all those years, it is resilience. The infamous tale always ended with the phoenix rising from its own ashes. However, the challenge now is in making this rise more sustainable, and hopefully making the 8^th^ burning the last.

On the bright side, despite being caught in this turmoil, Lebanon still managed to update its national climate pledge under the Paris Agreement [16], committing to increasing its Greenhouse Gas emission reduction target, increasing the percentage of the country’s electrical and heating demands from renewable energy sources by 2030, and synchronising overall with the 2030 Agenda for Sustainable Development. In fact, the current electricity shortage and power crisis had driven people faster towards renewable energy, not out of environmental awareness, but rather out of dire need [17]. In the recent years since the crisis, there was a significant move towards green energy by both the private and public sector, evidenced by a record high demand for photovoltaic systems. This is despite the fact that financing schemes, available pre 2019 as ‘green loans’ to end-users, are no longer available and the purchases are only possible through cash payments [17]. In addition, the crisis of 2019, coupled with the consecutive lockdowns imposed due to the COVID-19 pandemic in 2020 and 2021, has led to a change in characteristics and patterns within the transport sector [18]. For instance, within the country’s car-centric culture, there was a 73% drop of newly registered cars in 2020, compared to 2019. Authorities also removed subsidies on fuel imports, leading to a skyrocketing of gasoline prices [18], pushing people towards alternative modes of transportations, including public transport and carpooling.

On a less bright note, the financial recovery plan that is aiming to revive the economy and overcome short-term financial challenges, has a risk of affecting long-term climate-proofing efforts [19]. It includes putting efforts in developing the industrial sector, especially for several emissions intensive industries, including consumer goods, construction materials, and food processing [19]. In addition, it aspires to turn Lebanon into an oil and gas producing country, as these resources are likely to be available in its territorial waters. These efforts, among the existing fragile infrastructure and deeply rooted corruption, might affect the country’s climate pledge and targets majorly. However, the same aforementioned corruption and dysfunction have prevented any reform projects from happening before, and there is no proof that any significant change in governmental approaches will lead to the actual realisations of these projects in the near future. In a country where leaders have always worked for short-term monetary gains, projects with long-term benefits or mitigation efforts have rarely seen the light.

## Conclusion

In a multilevel crisis-torn country, it might be difficult to look under the microscope on the impact of climate change in particular on cancer care, without accounting for the other ongoing troubles. Political corruption leads to ongoing deteriorating infrastructure, inefficient policies, private sector exploiting the natural resources, increasing pollution to land, air, and sea, feeding into the climate changes; coupled with a financial crisis, increasing poverty, and drain of essential skilled workers, a crumbling healthcare system; Protecting the public health becomes a major challenge. In particular, protecting vulnerable populations such as cancer patients, becomes a greater challenge yet a priority. The healthcare system in Lebanon was able to adapt to change and retain its functionality throughout the different crises that faced the country, mainly due to the major networking efforts with multitude of partners within the health sector, and the international support and constant stream of funds received to alleviate the pressures of the refugees’ influx [4] and after the port explosion, for example. The adequate supply of health human resources is another vital element in the resilience of the healthcare system in Lebanon [4], therefore the dangers of the current exodus in shaking this resilience. The resilience of the system is currently being tested as the crisis evolves, and should be continuously monitored.

## Call for action

As the economy is being rebuilt, climate-proofing efforts should not be ignored. As food is being brought back on the table, it should be ensured that it is healthy food; as more jobs are created, they should be leaving less of a carbon footprint and honouring the climate pledge that Lebanon took; as the infrastructure and healthcare system of the country are being repaired, and made financially resilient, they should also be made climate-resilient to ensure the capability of providing care during upcoming climate-change related events. It is a crucial point in Lebanon’s modern history, it should be done right. A phoenix could rise from its own ashes, however a country might not be able to rise again if it was to be burnt by irreversible climate changes. No more grey ashes, only green recovery; act now.

## Author and affiliations

Mohamad Ibrahim, Clinical Research Nurse at the Royal Marsden Hospital, Fulham, London, UK.

The author has no current affiliations with any institution in Lebanon.

## Conflicts of interest

None to declare.

## Funding

None received.
